# P-2206. Impact of Sustained Virologic Response Achieved with Direct-Acting Antiviral Agents on Liver Fibrosis and Steatosis: A Systematic Review and Meta-Analysis

**DOI:** 10.1093/ofid/ofae631.2360

**Published:** 2025-01-29

**Authors:** Stephanos Vassilopoulos, Athanasios Vassilopoulos, Abby London, Markos Kalligeros, Eleftherios Mylonakis

**Affiliations:** Warren Alpert Medical School of Brown University, Rhode Island Hospital, Providence, RI, Providence, RI; Warren Alpert Medical School of Brown University, Rhode Island Hospital, Providence, RI, Providence, RI; Department of Medicine, Warren Alpert Medical School of Brown University, Rhode Island Hospital, Providence, Rhode Island; Warren Alpert Medical School of Brown University, Rhode Island Hospital, Providence, RI, Providence, RI; Houston Methodist Hospital, Houston, TX, Houston, Texas

## Abstract

**Background:**

HCV (hepatitis C virus) infection is a primary cause of liver cirrhosis and hepatocellular cancer. The association between viral eradication, defined as a sustained virologic response (SVR) 12-24-48 weeks post-treatment, and decline in liver steatosis and stiffness has not been elucidated.

Weighted mean difference of liver stiffness measurement between sustained virologic response in 12 weeks and baseline

**Figure:**
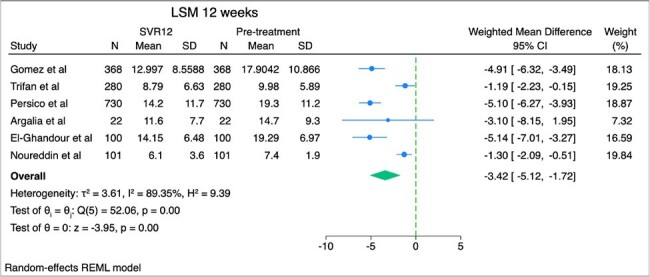

**Methods:**

We searched PubMed and EMBASE for studies that included patients with HCV infection who received direct acting antiviral agents (DAAs) with pre and post treatment measurements of liver fibrosis and steatosis. Liver fibrosis was evaluated with liver stiffness measurements (LSM) by utilizing vibration controlled transient elastography, while liver fat accumulation was quantified using controlled attenuation parameter (CAP). We present the weighted mean difference (WMD) and 95% confidence intervals (CI) of LSM and CAP with random-effects meta-analysis between pretreatment and SVR (at 12, 24 and 48 weeks).

Weighted mean difference of liver stiffness measurement between sustained virologic response in 24 weeks and baseline

**Figure:**
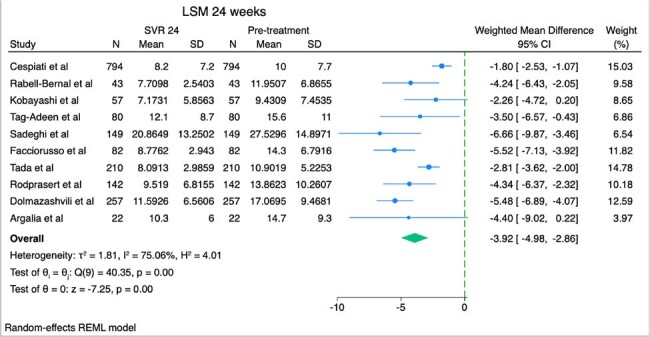

**Results:**

After screening 815 citations and excluding studies involving patients coinfected with HIV or those with a history of liver transplant, we identified and analyzed studies reporting LSM and CAP values before and after achieving SVR with DAA treatment. Specifically, 6 studies reported LSM at SVR 12 weeks, 10 studies at SVR 24 weeks, 11 studies at SVR 48 weeks, and 5 studies reported CAP at SVR 24 weeks. Among 1601 patients who achieved SVR at 12 weeks, LSM decreased by 3.42 kPa (95% CI, -5.12, -1.72). Among 1836 patients who achieved SVR at 24 weeks, LSM decreased by 3.92 kPa (95% CI, -4.98, -2.86). Among 1751 patients who achieved SVR at 48 weeks, LSM decreased by 4.37 kPa (95% CI, -5.60, -3.15). Among 1216 patients who achieved SVR at 24 weeks, CAP increased by 1.92 dB/m (95% CI, -2.42, 6.27), a result that was not statistically significant.

Weighted mean difference of liver stiffness measurement between sustained virologic response in 48 weeks and baseline

**Figure:**
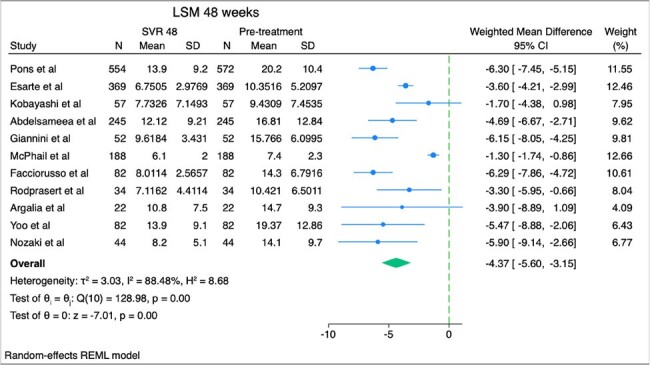

**Conclusion:**

We found that achieving SVR in patients with hepatitis C virus infection resulted in considerable reductions in liver stiffness measurements. Changes in steatosis values were not statistically significant, and additional prospective studies should assess for alterations in liver fat accumulation among patients with HCV infection achieving SVR.

**Disclosures:**

Eleftherios Mylonakis, MD, PhD, BARDA: Grant/Research Support|Basilea: Advisor/Consultant|Chemic Labs/KODA Therapeutics: Grant/Research Support|Cidara: Grant/Research Support|Leidos Biomedical Research Inc./NCI: Grant/Research Support|NIH SciClone Pharmaceuticals: Grant/Research Support|NIH/NIAID: Grant/Research Support|NIH/NIGMS: Grant/Research Support|Pfizer: Grant/Research Support|Regeneron Pharmaceuticals, Inc.: Grant/Research Support

